# Relative Leukocyte Telomere Length and Genetic Variants in Telomere-Related Genes and Serum Levels Role in Age-Related Macular Degeneration

**DOI:** 10.3390/cells11233847

**Published:** 2022-11-30

**Authors:** Alvita Vilkeviciute, Greta Gedvilaite, Mantas Banevicius, Loresa Kriauciuniene, Dalia Zaliuniene, Olivija Dobiliene, Rasa Liutkeviciene

**Affiliations:** 1 Laboratory of Ophthalmology, Neuroscience Institute, Medical Academy, Lithuanian University of Health Sciences, LT-50161 Kaunas, Lithuania; 2 Department of Ophthalmology, Medical Academy, Lithuanian University of Health Sciences, LT-50161 Kaunas, Lithuania; 3 Department of Cardiology, Medical Academy, Lithuanian University of Health Sciences, LT-50161 Kaunas, Lithuania

**Keywords:** relative leukocyte telomere length, age-related macular degeneration, SNP, TERT, TERT-CLPTM1, TRF1, TRF2, TNKS2, TERF-1 and TERF2 levels

## Abstract

Telomere shortening is well known to be associated with ageing. Age is the most decisive risk factor for age-related macular degeneration (AMD) development. The older the individual, the higher the AMD risk. For this reason, we aimed to find any associations between telomere length, distribution of genetic variants in telomere-related genes (*TERT*, *TERT-CLPTM1*, *TRF1*, *TRF2*, and *TNKS2*), and serum TERF-1 and TERF2 levels on AMD development. Methods: Our study enrolled 342 patients with AMD and 177 healthy controls. Samples of DNA from peripheral blood leukocytes were extracted by DNA salting-out method. The genotyping of *TERT* rs2736098, rs401681 in *TERT-CLPTM1* locus, *TRF1* rs1545827, rs10107605, *TNKS2* rs10509637, rs10509639, and *TRF2* rs251796 and relative leukocyte telomere length (T/S) measurement were carried out using the real-time polymerase chain reaction method. Serum TERF-1 and TERF2 levels were measured by enzymatic immunoassay (ELISA). Results: We found longer telomeres in early AMD patients compared to the control group. Additionally, we revealed that minor allele C at *TRF1* rs10107605 was associated with decreases the odds of both early and exudative AMD. Each minor allele G at *TRF2* rs251796 and *TRF1* rs1545827 C/T genotype and C/T+T/T genotypes, compared to the C/C genotype, increases the odds of having shorter telomeres. Furthermore, we found elevated TERF1 serum levels in the early AMD group compared to the control group. Conclusions: In conclusion, these results suggest that relative leukocyte telomere length and genetic variants of *TRF1* and *TRF2* play a role in AMD development. Additionally, TERF1 is likely to be associated with early AMD.

## 1. Introduction

Telomeres are nucleoprotein complexes that cap the ends of eukaryotic chromosomes [1]. These caps are constructed of short repetitive (TTAGGG)n nucleotide sequences with a single-stranded overhang and a protein complex, which are essential in maintaining the proper telomeric structure that protects chromosomes from end-to-end fusion, inappropriate DNA repair, atypical recombinations, and premature senescence [2,3]. Telomeric DNA length is regulated by the enzyme telomerase and six telomere-associated proteins—TRF1, TRF2, POT1, RAP1, TIN2, and TPP1 [4]. Telomerase adds the telomeric repeat sequence directly to the single-strand 3’ overhang. Additionally, it has been found that the catalytic subunit of telomerase, telomere reverse transcriptase (TERT), is the most crucial determinant in the regulation of telomerase expression [5]. Telomeric repeat binding factor 1 (TRF1) and telomeric repeat binding factor 2 (TRF2) are major telomere-related proteins that directly bind to the double-strand region of the telomere [6,7]. The TRF1 is a negative regulator of telomerase activity and increases telomere length when inhibited by the tankyrase (TNKS), a poly (ADP-ribose) polymerase (PARP), which can function as a positive regulator of telomere length in human cells [8]. The essential role of TRF2 in telomere end protection has been demonstrated. TRF2 protects chromosome ends mainly by the invasion of the 3′ single-stranded overhang into the duplex telomeric repeats, thus forming protective structures known as t-loops, which prevent DNA ends from being recognized by the DNA damage response and telomerase [2,9]. TRF1-interacting protein 2 (TIN2) is also one of the critical components of the telosome and bridges TRF1 and TRF2 with TPP1 that recruits telomerase to telomeres and enhances telomerase processivity upon complexation with another principal telomere-related protein protection of telomeres 1 (POT1) [10,11]. TPP1 provides the stability of POT1, and the TPP1-POT1 complex prevents a DNA damage repair response at the telomeric overhang site [12,13,14]. Repressor/activator protein 1 (RAP1) is recruited to telomeric repeats by TRF2. It ensures genome stability by protecting telomeric DNA ends from non-homologous end joining and from homologous recombination that can alter telomere length [15,16,17].

On the other hand, determinants of telomere length are still not well known. While at birth, telomere length ranges from 0.15 to 50 kilobases (kb), but it dramatically decreases during the lifespan; it is estimated that about 50 base pairs are lost during each cell division [18,19]. Age-associated telomere shortening is mainly associated with normal cellular ageing and human senescence [20,21]. When telomeres critically shorten, cell proliferation halts and the cells become senescent [22,23]. Telomere erosion has also been associated with inflammatory processes, oxidative stress, and several lifestyle factors [24,25,26,27], leading to cancer development. While it has been estimated that 80–90% of all cancers depend on telomerase for their unlimited proliferative capacity [28], some studies show controversial results, demonstrating shorter telomeres in cancer cohorts compared to healthy controls [29].

Telomeres also play a crucial role in age-related diseases [30,31,32], such as age-related macular degeneration (AMD) [33]. An association has been found between the geographic atrophy (GA) subtype of AMD and leukocyte telomere length (LTL) [33]. GA is described as the late stage of AMD, leading to irreversible vision loss [34]. Its prevalence increases with age [35], and adequate medical treatment is still not found.

We conducted a case–control study of Lithuanian subjects to evaluate associations between relative leukocyte telomere length, genetic variants in telomere-related genes (*TERT* rs2736098, rs401681 in *TERT*-*CLPTM1* locus, *TRF1* rs1545827, rs10107605, *TNKS2* rs10509637, rs10509639, and *TRF2* rs251796), and serum TERF-1 and TERF2 levels on the risk of age-related macular degeneration development.

## 2. Materials and Methods

Our study was conducted at the Department of Ophthalmology, Hospital of Lithuanian University of Health Sciences, and in the Laboratory of Ophthalmology, Neuroscience Institute, Lithuanian University of Health Sciences. Ethical approval was obtained from the Ethics Committee for Biomedical Research (No: BE-2-/48).

Ophthalmological evaluation, study group formation, DNA extraction, genotyping, and relative leukocyte telomere length measurement methods were described in detail in our previous studies [36,37].

### 2.1. Control Group Justification

Control group I do not differ from Early AMD group (*p* > 0.05), but is statistially significantly younger than Exudative AMD group (*p* < 0.05), so the Control group II was formed.

Control group II was formed using the same subjects from Control group I, only the younger subjects were excluded to ensure that there is no statistically significant difference between Exudative AMD and Control group II (*p* > 0.05).

### 2.2. Serum Protein Measurement

Serum TERF-1 and TERF2 levels were measured by enzymatic immunoassay (ELISA). The detection range of the TERF1 ELISA kit was 0.156 ng/mL–10 ng/mL, with a minimum detectable level of 0.094 ng/mL. TERF2 detectable range was 0.16–10 ng/mL, and the minimum level was 0.10 ng/mL. ELISA was performed as described in the instructions for TERF1 and TERF2 ELISA kits (Thermo Fisher Scientific Inc., Vienna, Austria). Absorbance was measured at 450 nm in a microplate reader (Multiskan Fc, Thermo Fisher Scientific Inc., Shanghai, China). A linear model was used to generate the standard curve, and the results were obtained after multiplication by the dilution factor (5×).

### 2.3. Statistical Analysis

Statistical analysis was performed using the SPSS/W 27.0 software (Statistical Package for the Social Sciences for Windows, Inc., Chicago, IL, USA). The data were presented as median with interquartile range (IQR) for continuous data. The normality of data distribution was checked using the Shapiro–Wilk test. For non-normally distributed data, the Mann–Whitney test was used to compare the data between two groups. For normally distributed data, the Pearson Chi-Square test was used to compare the data between two groups. Differences were considered statistically significant when *p* < 0.05. Bonferroni correction was not used in regard to 2 SNPs per gene that were analysed.

Frequencies of genotypes and alleles, and gender are reported using absolute numbers with percentages in brackets. The distributions of the genotypes and alleles, male and female distributions between study groups were compared using the χ^2^ test. Binomial logistic regression analysis was performed to estimate the impact of genotypes on early, exudative AMD development and presented as odds ratios with its 95% confidence intervals.

The best genetic model selection was based on the Akaike Information Criterion (AIC); therefore, the best genetic models were those with the lowest AIC values.

## 3. Results

### 3.1. Demographic Characteristics

The genotyping and relative leukocyte telomere length (T/S) were analyzed in 177 patients with early AMD and 165 exudative AMD patients. Because age is associated with telomere length, two control groups formed: age-matching control group I (*n* = 177) for early AMD analysis and control group II (*n* = 118) for exudative AMD. All demographic characteristics are presented in Table 1.

### 3.2. Relative Leukocyte Telomere Length

**Figure 1:**
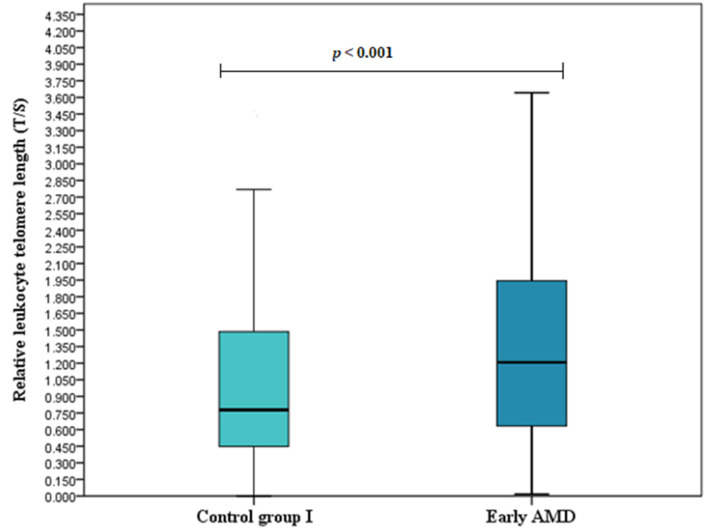
Relative leukocyte telomere length in early AMD patients and the control group I. Relative leukocyte telomere length (T/S) in early AMD patients versus healthy controls are presented as box-and-whisker plots with the median and IQR. Mann–Whitney U test was used to assess T/S differences between patients with early AMD and control groups; *p* < 0.001.

**Figure 2:**
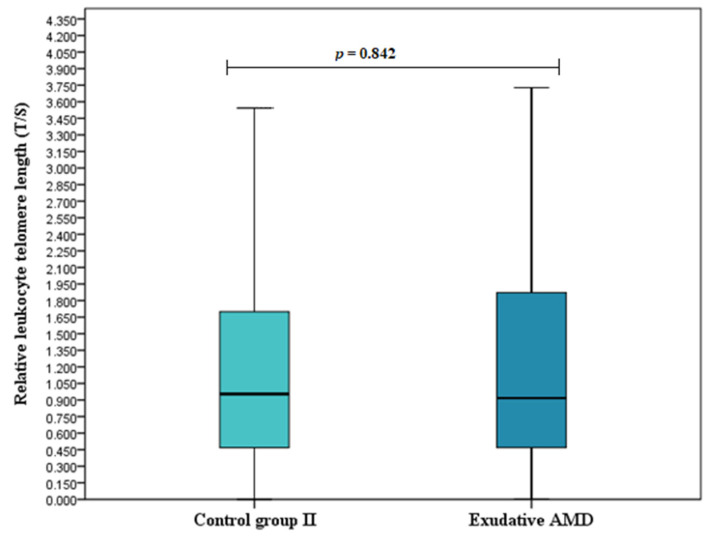
Relative leukocyte telomere length in exudative AMD patients and the control group II. Relative leukocyte telomere length (T/S) in exudative AMD patients versus healthy controls are presented as box-and-whisker plots with the median and IQR. Mann–Whitney U test assessed T/S differences between patients with exudative AMD and control groups; *p* = 0.842.

**Figure 3:**
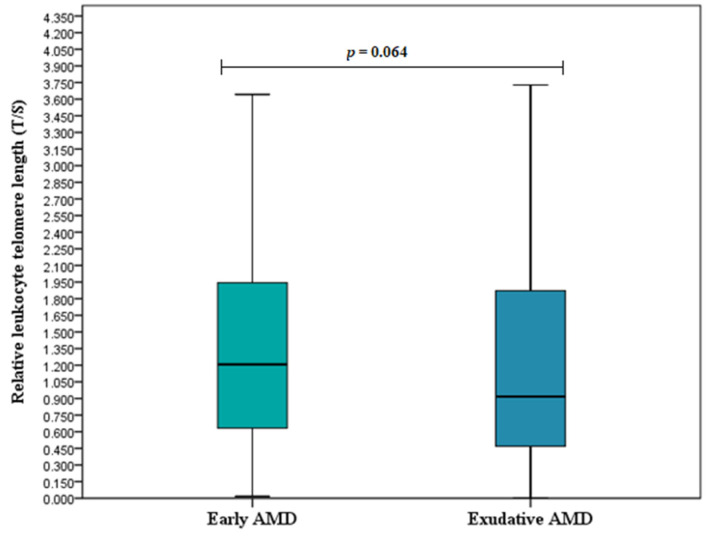
Relative leukocyte telomere length in early and exudative AMD patients. Relative leukocyte telomere length (T/S) in early AMD patients versus exudative AMD patients are presented as box-and-whisker plots with the median and IQR. Mann–Whitney U test was used to assess the differences in T/S between patients with early and exudative AMD; *p* = 0.064.

Relative leukocyte telomere length (T/S) was determined for all study subjects and compared between study groups. We found longer telomeres in early AMD patients compared to the control group I (T/S (median (IQR): 1.207 (1.319) vs. 0.778 (1.057), respectively, *p* < 0.001) (Figure 1). No differences were observed between exudative AMD patients and the control group II: (T/S (median (IQR): 0.918 (1.406) vs. 0.954 (1.239), respectively, *p* = 0.842) (Figure 2) or between early and exudative AMD patients (T/S (median (IQR): 1.207 (1.319) vs. 0.918 (1.406), respectively, *p* = 0.064) (Figure 3).

### 3.3. The Genotyping of TERT rs2736098, rs401681, TRF1 rs1545827, rs10107605, TNKS2 rs10509637, rs10509639 and TRF2 rs251796

Hardy–Weinberg equilibrium (HWE) was evaluated for all SNPs in both control group I and control group II. Only one SNP *TRF1* rs10107605 did not follow the HWE, which might be caused by low sample size in study groups.

The genotype and allele frequencies of *TERT* rs2736098, rs401681, *TRF1* rs1545827, rs10107605, *TNKS2* rs1050963, rs10509639, and *TRF2* rs251796 in the early, exudative AMD and control groups are shown in Table 2. Statistical analysis was performed to compare the genotype and allele frequencies between the early AMD group and the control group I as well as between the exudative AMD group and the control group II. We found that *TRF1* rs10107605 minor allele C was statistically significantly less frequent in early AMD patients than in control group I subjects: 8.5% vs. 14.1%, respectively, *p* = 0.018 (Table 2). Additionally, we revealed that *TRF1* rs10107605 genotypes (A/A, A/C, and C/C) distribution differed statistically significantly between exudative AMD and control group II subjects: 87.1%, 12.9%, and 0% vs. 78.8%, 14.4% and 6.8%, respectively, *p* = 0.004, and the minor allele C was less frequent in exudative AMD patients than in control group II subjects: 6.5% vs. 14.0%, respectively, *p* = 0.003 (Table 3).

Binomial logistic regression was performed to evaluate the impact of *TERT* rs2736098, rs401681, *TRF1* rs1545827, rs10107605, *TNKS2* rs1050963, rs10509639 and *TRF2* rs251796 on early and exudative AMD development. Our results revealed that genotype CC at rs10107605 was associated with about 75% decreased odds of early AMD development under the codominant and recessive models (OR = 0.251; 95% CI: 1.333–3.870; *p* = 0.037 and OR = 0.260; 95% CI: 1.483–3.970; *p* = 0.041, respectively). Additionally, the analysis showed that minor allele C was associated with decreased odds of early and exudative AMD as well (OR = 0.632; 95% CI: 1.333–3.870; *p* = 0.038 and OR = 0.490; 95% CI: 1.483–3.970; *p* = 0.010, respectively) (Table 4).

The leukocyte telomeres were divided into short and long telomeres by all study subjects’ median telomere length. Statistical analysis was performed to compare the genotype and allele frequencies between the two groups. Statistically significant differences were found only by comparing genotype and allele frequencies of *TRF2* rs251796 between the long and short telomere groups (*p* = 0.043 and *p* = 0.011, respectively) (Table 5). The binomial logistic regression analysis revealed that G/G genotype compared to A/A genotype was associated with 2-fold increased odds of having short telomeres (OR = 2.039; 95% CI: 1.050–3.961; *p* = 0.035); A/G+G/G genotypes carriers compared to A/A genotype had 1.5-fold increased odds of having short telomeres (OR = 1.499; 95% CI: 1.056–2.128; *p* = 0.023), and overall each allele G at *TRF2* rs251796 was associated with 1.4-fold increased odds of having short telomeres (OR = 1.418; 95% CI: 1.078–1.866; *p* = 0.013) (Table 6). Additionally, we determined that *TRF1* rs1545827 C/T genotype and C/T+T/T genotypes compared to C/C genotype carriers were associated with 1.6 and 1.5-fold increased odds of having short telomeres under the codominant and dominant models (OR = 1.555; 95% CI: 1.055–2.293; *p* = 0.026 and OR = 1.518; 95% CI: 1.054–2.186; *p* = 0.025, respectively) (Table 6).

**Figure 4:**
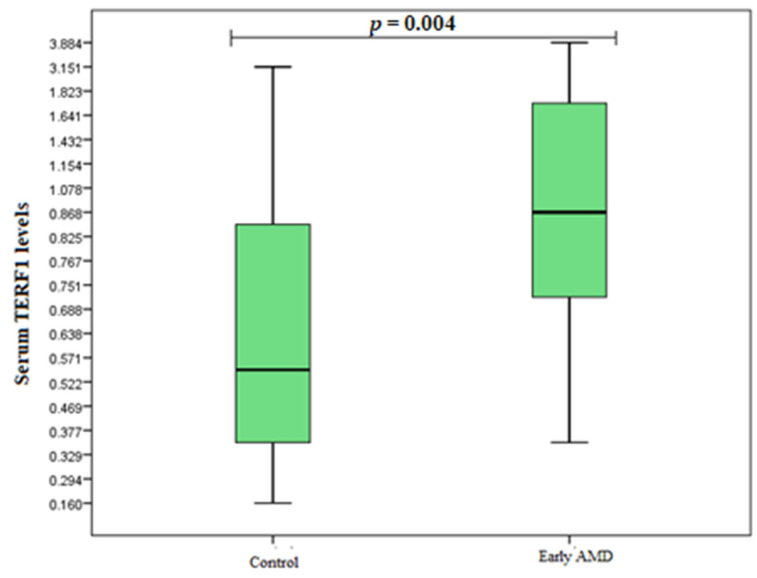
Serum levels of TERF1 in patients with early AMD and control group. AMD: age-related macular degeneration; Serum protein values are presented as median and IQR. Mann–Whitney U test was used to assess serum TERF1 levels differences between patients with early AMD and control groups; *p* = 0.004.

**Figure 5:**
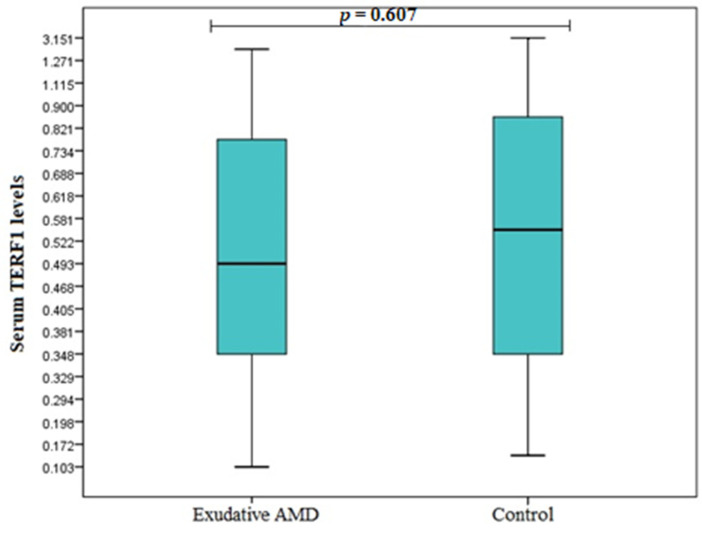
Serum levels of TERF1 in patients with exudative AMD and control group. AMD: age-related macular degeneration; Serum protein values are presented as median and IQR. Mann–Whitney U test was used to assess serum TERF1 levels differences between patients with exudative AMD and control groups; *p* = 0.607.

**Figure 6:**
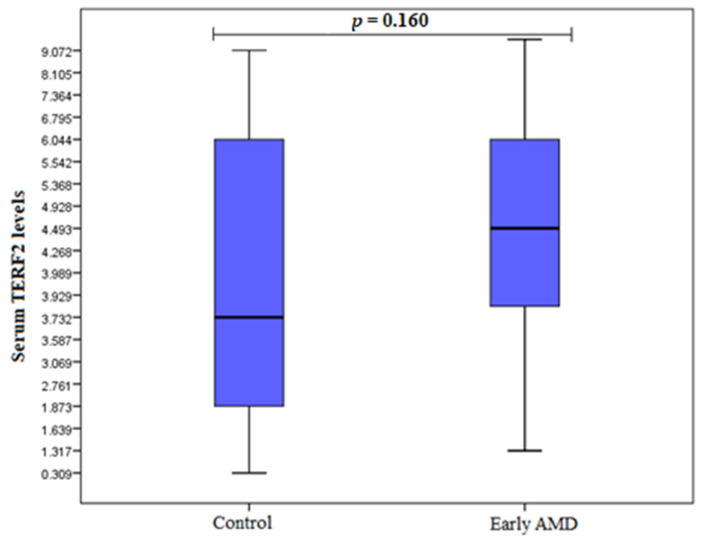
Serum levels of TERF2 in patients with early AMD and control group. AMD: age-related macular degeneration; Serum protein values are presented as median and IQR. Mann–Whitney U test was used to assess serum TERF2 levels differences between patients with early AMD and control groups; *p* = 0.160.

**Figure 7:**
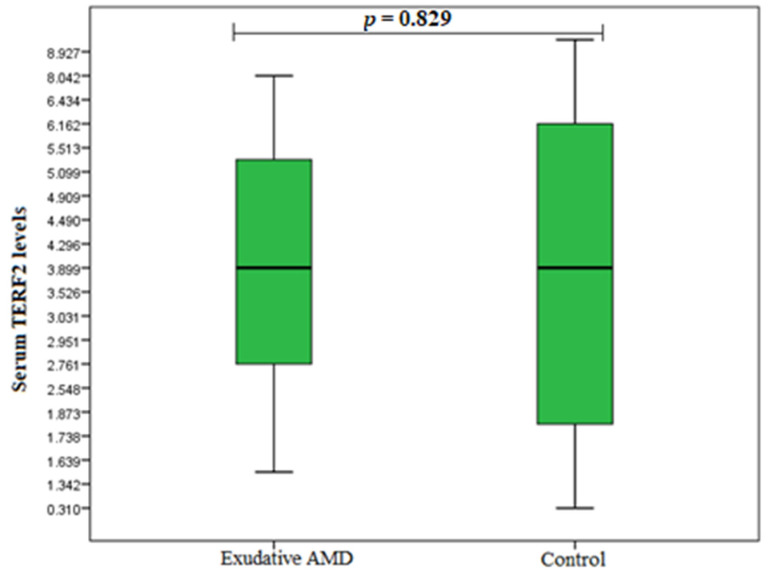
Serum levels of TERF2 in patients with exudative AMD and control group. AMD: age-related macular degeneration; Serum protein values are presented as median and IQR. Mann–Whitney U test was used to assess serum TERF2 levels differences between patients with exudative AMD and control groups; *p* = 0.004.

TERF1 and TERF2 serum levels were measured in duplicates for 20 early AMD patients, 20 exudative AMD patients, and 20 control subjects. Analysis showed elevated TERF1 serum levels in the early AMD group compared to control subjects (median (IQR): 0.850 (1.025) ng/mL vs. 0.546 (0.526) ng/mL, *p* = 0.004) (Figure 4). However, there were no statistically significant differences between exudative AMD and control groups (median (IQR): 0.493 (0.459) ng/mL vs. 0.546 (0.526) ng/mL, *p* = 0.607) (Figure 5). Additionally, no statistically significantly results were found analysing TERF2 serum levels between early AMD vs. control group, and exudative AMD vs. control group (median (IQR): 4.476 (2.200) ng/mL vs. 3.743 (4.235) ng/mL, *p* = 0.160 (Figure 6); 3.911 (2.462) ng/mL vs. 3.743 (4.235) ng/mL, *p* = 0.829 (Figure 7), respectively).

## 4. Discussion

The discovery and explanation of telomere complex role in biology warranted the 2009 Nobel Prize in medicine [38]. Understanding basic biological mechanisms and the emerging impact of telomerase and telomere biology in medicine provides a unique opportunity to study age-related diseases such as age-related macular degeneration.

Many studies are analyzing TL association with ageing, and results are bewildering and wondrous. Some studies have proved that LTL declines by 0.003 ln(T/S) per year on average from the age of 50 to 90 [33,39], while other studies have stated that there is no significant association between the shortened LTL and ageing [40,41]. Muezzinler and colleagues have determined that a reduction in LTL is approximately 0.5/(10 years) in cohorts of various age groups [42]. For example, in a Japanese study, Arai and colleagues have concluded that inflammation is a crucial malleable driver of ageing up to extreme old age in humans. Still, telomere length is not a predictor of successful ageing in centenarians and semi-supercentenarians [43]. Another study has stated that individuals with shorter telomeres are characterized by a higher mortality rate, nearly twice as high as those with longer telomeres [44]. A study conducted by Mons et al. [45] analyzed more than 12,000 subjects of two population-based studies (ESTHER and Nurses’ Health Study) and identified that subjects with shorter telomeres (1st quintile) have a higher hazard ratio for all-cause mortality (1.66, 95% CI 1.09–2.53, *p* = 0.018) compared to those with longer telomeres (5th quintile), in agreement with the study conducted by Goglin et al. [44]. 

The first study (2015) analyzing the exudative AMD association with LTL was conducted on the Han Chinese population [33]. The researchers analyzed 197 AMD cases (both exudative and atrophic: 76 GA cases (38.57%), 52 CNV cases (26.40%), and 69 advanced AMD cases lacking further subtype information (35.03%)) and 259 healthy controls. Scientists revealed a strong association between AMD and LTL (OR = 2.24; 95% CI = 1.68–3.07; *p* = 0.0001) after adjustment for age and sex. Furthermore, their results showed a significant association between the GA subtype and the LTL (OR = 4.81; 95% CI = 3.15–7.82; *p* = 0.0001), also after adjustment for age and sex. They proved that LTL plays a role in AMD’s pathological mechanisms, mainly in the GA subtype but not in the CNV [33]. Meanwhile, another study by Immonen et al. [39] did not reveal any statistically significant results comparing the mean (SD) telomere length in AMD patients (0.68) and the control group (0.69) (*p* = 0.485). The corresponding proportions of telomeres <5° kb were 10.60 (2.76) and 10.05 (2.64) (*p* = 0.197). In this study, a hundred (82.6%) patients had neovascular AMD in the worse eye, seventeen (14%) large drusen, and four (3.3%) central geographic atrophy. There were no differences in the telomere length between patients with drusen or exudative AMD [39]. Two studies (Immonen et al. [39] and Weng, X. et al. [33]) have presented different results. Still, the difference between the studies could be explained by the different proportions of AMD subtypes among the studied AMD patients. In Immonen et al.’s study, AMD patients had mostly neovascular AMD, and only 3% had geographic atrophy AMD. In the other study, Weng, X. et al. evaluated 76 GA cases (38.57%) and 52 CNV cases (26.40%), comparing them with 259 healthy individuals separately. Differences in telomere length could be due to the phenotypic difference between GA and CNV.

Several diseases are related to the short telomere length: ataxia-telangiectasia, Bloom syndrome, dyskeratosis congenital, Fanconi anaemia, Nijmegen breakage syndrome, Werner syndrome [46], and idiopathic pulmonary fibrosis [47]. Short telomere length is also associated with other common age-related diseases: coronary artery disease [48], chronic obstructive pulmonary disease [49], osteoporosis [50], and Alzheimer’s disease [51,52], as well as AMD [33,39].

It has been thought that the leukocyte telomere length may reflect the systemic telomerase capacity of an individual. Alternatively, shortened leukocyte telomeres may be associated with the chronic activation of the immune system beyond the reparative capacity of telomerase [53]. Such chronic systemic inflammation has been reported in the pathogenesis of cardiovascular disease and AMD [54]. 

Drigeard Desgarnier et al. found telomere length differences in different human eye structures [55]. Moreover, Bell et al. observed a unique telomere DNA expansion phenotype in the rod cells but not in other retinal cells [56].

Analysing the AMD pathogenesis, scientists suggested that the senescence of the RPE cells might play a role in AMD development [57] through several pathways, including oxidative stress response [58]. Oxidative stress damages telomeres due to their guanine-rich DNA structure. While it can be more challenging to repair, in some cases, the telomerase may extend oxidative stress-shortened telomeres, preventing further RPE cell degeneration following AMD progression [59].

In the experimental model, telomere shortening inhibited neovascularization [60]. It is possible that telomere shortening might have a role in the pathogenesis of geographic atrophy. Unfortunately, the number of patients with geographic atrophy was too small (*n* = 4 and *n* = 76 [33,40]) that this hypothesis could not be approved; besides, it was drawn in different populations (Chinese and European (Finland)). It is also stated that telomere length does not correlate with mortality and morbidity in the very old [61]. 

However, normal age-related shortening of telomeres may still be one of the ageing changes that make conditions favourable for the action of specific pathogenic factors of AMD development. 

Based on Mendelian randomization approaches, a score built from SNPs associated with LTL was suggested as a critical risk marker (“teloscore” which explains 2.2% of the telomere variability) [62]. Moreover, genome-wide association and candidate gene studies have shown that SNPs in the *TERT* gene and telosome complex genes were associated mostly with cancer risk. While it has been hypothesized that polymorphisms in the *TERT* gene might be related to cancer via their effects on the expression of TERT, others have revealed the associations between *TERT* polymorphisms and increased TERT transcription activity [63,64]. Controversial results have been published, showing differences among the types of cancer [65,66]. Furthermore, a meta-analysis has revealed an association between the allele A at *TERT* rs2736098 G > A (located on 5p15.33) and cancer development. Besides, the ethnicity-specific effect has also been found while analyzing different subgroups [67]. Another meta-analysis has shown that allele C at *TERT*-*CLPTM1L* rs401681 (located on 5p15.33) was a low-penetrance risk allele for the development of lung, bladder, prostate cancers, and basal cell carcinoma but also a potential protective allele for melanoma and pancreatic cancer [68]. Additionally, the *TRF2* rs251796 was significantly associated with lung cancer as well [69]. No other experiments were performed in eye disease studies with SNPs, which could be compared to our results. Our study revealed that the *TRF1* rs10107605 was associated with decreased odds of early and exudative AMD development, while the *TRF2* rs251796 and *TRF1* rs1545827 variants were linked to shorter telomeres.

No other study has investigated the link between AMD and TRF1/TRF2 expression. However, only one study has been conducted on the association between TRF1/TRF2 and TL-shortening disease [52]. Wu et al. reported that the expression of TRF1 was elevated in Alzheimer’s patients than in the control group, while the TRF2 expression was significantly lower than in control subjects. This investigation showed that TRF1 and TRF2 could be associated with TL-shortening and may be closely connected with Alzheimer’s disease progression [70]. Several authors examined TRF1/TRF2 expression in many types of cancers. In the study by Shi et al., the link between the expression of TRF1 protein and human leukaemia was analyzed. The study showed that TRF1 protein concentration was lower in leukaemia patients than in control subjects [71]. However, Lin and other authors reported that the expression of TRF1 was significantly lower in lung cancer tissues than in normal tissues; no significant differences were found between TRF2 [72]. Our study revealed that the expression of TRF1 in serum was elevated in the early AMD group compared to control subjects. While our study revealed significant results, it is still important to highlight that further studies with larger sample size are necessary to confirm such associations. Moreover, other AMD risk factors should be included into future studies.

## 5. Conclusions

Our study revealed longer telomeres in early AMD patients compared to the control group (T/S (median (IQR): 1.207 (1.319) vs. 0.778 (1.057), respectively, *p* < 0.001). Additionally, TRF2 rs251796 and TRF1 rs1545827 variants were linked to shorter telomeres, while TRF1 rs10107605 was associated with decreased odds of early and exudative AMD development. We also found elevated TERF1 serum levels in the early AMD group compared to the control group (*p* = 0.004). 

## Figures and Tables

**Table 1:** Demographic characteristics of patients with early and exudative age-related macular degeneration (AMD) and control group subjects.

Characteristic	Early AMD Group *n* = 177	Control Group I *n* = 177	*p*-Value	Exudative AMD Group *n* = 165	Control Group II *n* = 118	*p*-Value
Gender, *n* (%)			0.002 *			0.121 *
Males	79 (28.8)	79 (44.6)		54 (34.8)	52 (44.1)	
Females	126 (71.2)	98 (55.4)		101 (65.2)	66 (55.9)	
Age, median (IQR), years	70 (14)	73 (13)	0.104 **	77 (10)	74 (11)	0.081 **

* Pearson Chi-Square; ** Mann–Whitney U test.

**Table 2:** Frequencies of genotypes and alleles of *TERT* rs2736098, rs401681, *TRF1* rs1545827, rs10107605, *TNKS2* rs10509637, rs10509639 and *TRF2* rs251796 in patients with early age-related macular degeneration (AMD) and the control group.

Polymorphism	Control Group I	HWE * *p* Value	Early AMD	* *p* Value
***TERT*** **rs2736098**				
C/C	98 (55.4)	0.213	101 (57.1)	0.837
C/T	63 (35.6)		63 (35.6)	
T/T	16 (9.0)		13 (7.3)	
Total	177 (100)		177 (100)	
C	259 (73.2)		265 (74.9)	0.607
T	95 (26.8)		89 (25.1)	
***TERT*** **rs401681**				
C/C	62 (35.0)	0.647	63 (35.6)	0.976
C/T	83 (46.9)		81 (45.8)	
T/T	32 (18.1)		33 (18.6)	
Total	177 (100)		177 (100)	
Allele				1.000
C	207 (58.5)		207 (58.5)	
T	147 (41.5)		147 (41.5)	
***TRF1*** **rs1545827**				
C/C	67 (37.9)	0.969	65 (36.7)	0.517
C/T	84 (47.5)		78 (44.1)	
T/T	26 (14.7)		34 (19.2)	
Total	177 (100)		177 (100)	
Allele				0.443
C	218 (61.6)		208 (58.8)	
T	136 (38.4)		146 (41.2)	
***TRF1*** **rs10107605**				
A/A	138 (78.0)	<0.001	150 (84.7)	0.068
A/C	28 (15.8)		24 (13.6)	
C/C	11 (6.2)		3 (1.7)	
Total	177 (100)		177 (100)	
Allele				0.018
A	304 (85.9)		324 (91.5)	
C	50 (14.1)		30 (8.5)	
***TNKS2*** **rs10509637**				
A/A	119 (67.2)	0.913	125 (70.6)	0.539
A/G	52 (29.4)		49 (27.7)	
G/G	6 (3.4)		3 (1.7)	
Total	177 (100)		177 (100)	
Allele				0.366
A	290 (81.9)		299 (84.5)	
G	64 (18.1)		55 (15.5)	
***TNKS2*** **rs10509639**				
A/A	147 (83.1)	0.737	153 (86.4)	0.451
A/G	29 (16.4)		24 (13.6)	
G/G	1 (0.6)		0 (0)	
Total	177 (100)		177 (100)	
Allele				0.326
A	323 (91.2)		330 (93.2)	
G	31 (8.8)		24 (6.8)	
***TRF2*** **rs251796**				
A/A	89 (50.3)	0.445	94 (53.1)	0.624
A/G	70 (39.5)		70 (39.5)	
G/G	18 (10.2)		13 (7.3)	
Total	177 (100)		177 (100)	
Allele				0.405
A	248 (70.1)		258 (72.9)	
G	106 (29.9)		96 (27.1)	

AMD: age-related macular degeneration; * χ^2^ test.

**Table 3:** Frequencies of genotypes and alleles of *TERT* rs2736098, rs401681, *TRF1* rs1545827, rs10107605, *TNKS2* rs10509637, rs10509639 and *TRF2* rs251796 in patients with exudative age-related macular degeneration (AMD) and the control group.

Polymorphism	Control Group II	HWE * *p* Value	Exudative AMD	* *p* Value
***TERT*** **rs2736098**				
C/C	63 (53.4)	0.881	89 (57.4)	0.518
C/T	46 (39.0)		59 (38.1)	
T/T	9 (7.6)		7 (4.5)	
Total	118 (100)		155 (100)	
C				0.340
T	172 (72.9)		237 (76.5)	
***TERT*** **rs401681**				
C/C	43 (36.4)	0.574	59 (38.1)	0.804
C/T	54 (45.8)		73 (47.1)	
T/T	21 (17.8)		23 (14.8)	
Total	118 (100)		155 (100)	
Allele				0.587
C	140 (59.3)		191 (61.6)	
T	96 (40.3)		119 (38.4)	
***TRF1*** **rs1545827**				
C/C	46(39.0)	0.743	53 (34.2)	0.672
C/T	54 (45.8)		74 (47.7)	
T/T	18 (15.3)		28 (18.1)	
Total	118 (100)		155 (100)	
Allele				0.310
C	146 (61.9)		180 (58.1)	
T	90 (38.1)		130 (41.9)	
***TRF1*** **rs10107605**				
A/A	93 (78.8)	<0.001	135 (87.1)	**0.004**
A/C	17 (14.4)		20 (12.9)	
C/C	8 (6.8)		0 (0.0)	
Total	118 (100)		155 (100)	
Allele				**0.003**
A	203 (86.0)		290 (93.5)	
C	33 (14.0)		20 (6.5)	
***TNKS2*** **rs10509637**				
A/A	82 (69.5)	0.356	106 (68.4)	0.957
A/G	31 (26.3)		43 (27.7)	
G/G	5 (4.2)		6 (3.9)	
Total	118 (100)		155 (100)	
Allele				0.911
A	195 (82.6)		255 (82.3)	
G	41 (17.4)		55 (17.7)	
***TNKS2*** **rs10509639**				
A/A	100 (84.7)	0.770	129 (83.2)	0.456
A/G	17 (14.4)		26 (16.8)	
G/G	1 (0.8)		0 (0.0)	
Total	118 (100)		155 (100)	
Allele				0.887
A	217 (91.9)		284 (91.6)	
G	19 (8.1)		26 (8.4)	
***TRF2*** **rs251796**				
A/A	60 (50.8)	0.685	83 (53.5)	0.855
A/G	47 (39.8)		60 (38.7)	
G/G	11 (9.3)		12 (7.7)	
Total	118 (100)		155 (100)	
Allele				0.581
A	167 (70.8)		226 (72.9)	
G	69 (29.2)		84 (27.1)	

AMD: age-related macular degeneration; * χ^2^ test.

**Table 4:** Binomial logistic regression analysis of TRF1 rs10107605 in patients with early and exudative AMD.

Model	Genotype/Allele	* OR (95% CI)	*p*	AIC
***Early AMD***				
***TRF1*** **rs10107605**				
**Codominant**	**A/C vs. A/A** **C/C vs. A/A**	0.789 (0.436–1.426) 0.251 (0.069–0.918)	0.432 **0.037**	489.080
**Recessive**	**C/C vs. A/A+A/C**	0.260 (0.071–0.949)	**0.041**	487.700
**Additive**	**C**	0.632 (0.410–0.974)	**0.038**	488.234
***Exudative AMD***				
***TRF1*** **rs10107605**				
**Additive**	**C**	0.490 (0.285–0.844)	0.010	368.362

AMD: age-related macular degeneration; OR: odds ratio; CI: confidence interval; *p* value: significance level (alpha = 0.05); AIC: Akaike information criterion; * OR was adjusted by gender in early AMD.

**Table 5:** Frequencies of genotypes and alleles of *TERT* rs2736098, rs401681, *TRF1* rs1545827, rs10107605, *TNKS2* rs10509637, rs10509639 and *TRF2* rs251796 in the long and short telomere groups (T/S median = 0.98961).

Polymorphism	Long Telomeres	Short Telomeres	* *p* Value
***TERT*** **rs2736098**			
C/C	145 (57.3)	143 (56.3)	0.969
C/T	91 (36.0)	93 (36.3)	
T/T	17 (6.7)	18 (7.1)	
Total	253 (100)	254 (100)	
Allele			
C	381 (75.3)	379 (74.6)	0.800
T	125 (24.7)	129 (25.4)	
***TERT*** **rs401681**			
C/C	91 (36.0)	92 (36.2)	0.754
C/T	115 (45.5)	121(47.6)	
T/T	47 (18.6)	41 (16.1)	
Total	253 (100)	254 (100)	
Allele			0.663
C	297 (58.7)	305 (60.0)	
T	209 (41.3)	203 (40.0)	
***TRF1*** **rs1545827**			
C/C	104 (41.1)	80 (31.5)	0.075
C/T	107 (42.3)	128 (50.4)	
T/T	42 (16.6)	46 (18.1)	
Total	253 (100)	254 (100)	
Allele			0.071
C	315 (62.3)	288 (56.7)	
T	191 (37.7)	220 (43.3)	
***TRF1*** **rs10107605**			
A/A	215 (85.0)	206 (81.1)	0.400
A/C	33 (13.0)	39 (15.4)	
C/C	5 (2.0)	9 (3.5)	
Total	253 (100)	254 (100)	
Allele			0.146
A	463 (91.5)	451 (88.8)	
C	43 (8.5)	57 (11.2)	
***TNKS2*** **rs10509637**			
A/A	172 (68.0)	176 (69.3)	0.895
A/G	74 (29.2)	70 (27.6)	
G/G	7 (2.8)	8 (3.1)	
Total	253 (100)	254 (100)	
Allele			0.845
A	418 (82.6)	422 (83.1)	
G	88 (17.4)	86 (16.9)	
***TNKS2*** **rs10509639**			
A/A	210 (83.0)	217 (85.4)	0.420
A/G	43 (17.0)	36 (14.2)	
G/G	0 (0)	1 (0.4)	
Total	253 (100)	254 (100)	
Allele			0.550
A	463 (91.5)	470 (92.5)	
G	43 (8.5)	38 (7.5)	
***TRF2*** **rs251796**			
A/A	145 (57.3)	120 (47.2)	**0.043**
A/G	92 (36.4)	107 (42.1)	
G/G	16 (6.3)	27 (10.6)	
Total	253 (100)	254 (100)	
Allele			**0.011**
A	382 (75.5)	347 (68.3)	
G	124 (24.5)	161 (31.7)	

* χ^2^ test.

**Table 6:** Binomial logistic regression analysis of *TRF2* rs251796 and *TRF1* rs1545827 in telomere shortening.

Model	Genotype/Allele	* OR (95% CI)	*p*	AIC
*TRF2* rs251796				
**Codominant**	**A/G vs. A/A** **G/G vs. A/A**	1.405 (0.972–2.033) 2.039 (1.050–3.961)	0.071 **0.035**	700.512
**Dominant**	**A/G+G/G vs. A/A**	1.499 (1.056–2.128)	**0.023**	699.690
**Additive**	**G**	1.418 (1.078–1.866)	**0.013**	698.517
***TRF1*** **rs1545827**				
**Codominant**	**C/T vs. C/C** **T/T vs. C/C**	1.555 (1.055–2.293) 1.424 (0.855–2.371)	**0.026** 0.174	701.651
**Dominant**	**C/T+T/T vs. C/C**	1.518 (1.054–2.186)	**0.025**	699.775

OR: odds ratio; CI: confidence interval; *p* value: significance level (alpha = 0.05); AIC: Akaike information criterion.

## Data Availability

The data presented in this study are available on request from the corresponding author.

## References

[1] Wai L.K. (2004). Telomeres, telomerase, and tumorigenesis—A review. MedGenMed.

[2] Griffith J.D., Comeau L., Rosenfield S., Stansel R.M., Bianchi A., Moss H., de Lange T. (1999). Mammalian Telomeres End in a Large Duplex Loop. Cell.

[3] Lundblad V., Szostak J.W. (1989). A mutant with a defect in telomere elongation leads to senescence in yeast. Cell.

[4] Liu D., O’Connor M.S., Qin J., Songyang Z. (2004). Telosome, a Mammalian Telomere-associated Complex Formed by Multiple Telomeric Proteins. J. Biol. Chem..

[5] Blasco M.A. (2003). Mammalian telomeres and telomerase: Why they matter for cancer and aging. Eur. J. Cell Biol..

[6] Broccoli D., Smogorzewska A., Chong L., de Lange T. (1997). Human telomeres contain two distinct Myb-related proteins, TRF1 and TRF2. Nat. Genet..

[7] Chong L., van Steensel B., Broccoli D., Erdjument-Bromage H., Hanish J., Tempst P., de Lange T. (1995). A Human Telomeric Protein. Science.

[8] Smith S., de Lange T. (2000). Tankyrase promotes telomere elongation in human cells. Curr. Biol..

[9] Doksani Y., Wu J.Y., de Lange T., Zhuang X. (2013). Super-Resolution Fluorescence Imaging of Telomeres Reveals TRF2-Dependent T-loop Formation. Cell.

[10] Nandakumar J., Cech T.R. (2013). Finding the end: Recruitment of telomerase to telomeres. Nat. Rev. Mol. Cell Biol..

[11] Palm W., de Lange T. (2008). How shelterin protects mammalian telomeres. Annu. Rev. Genet..

[12] Hockemeyer D., Palm W., Else T., Daniels J.-P., Takai K.K., Ye J.Z.-S., Keegan C.E., de Lange T., Hammer G.D. (2007). Telomere protection by mammalian Pot1 requires interaction with Tpp1. Nat. Struct. Mol. Biol..

[13] Churikov D., Price C.M. (2008). Pot1 and cell cycle progression cooperate in telomere length regulation. Nat. Struct. Mol. Biol..

[14] Guo X., Deng Y., Lin Y., Cosme-Blanco W., Chan S., He H., Yuan G., Brown E.J., Chang S. (2007). Dysfunctional telomeres activate an ATM-ATR-dependent DNA damage response to suppress tumorigenesis. EMBO J..

[15] Bae N.S., Baumann P. (2007). A RAP1/TRF2 complex inhibits non-homologous end-joining at human telomeric DNA ends. Mol. Cell.

[16] Sarthy J., Bae N.S., Scrafford J., Baumann P. (2009). Human RAP1 inhibits non-homologous end joining at telomeres. EMBO J..

[17] Sfeir A., Kabir S., van Overbeek M., Celli G.B., de Lange T. (2010). Loss of Rap1 Induces Telomere Recombination in the Absence of NHEJ or a DNA Damage Signal. Science.

[18] Aubert G., Lansdorp P. (2008). Telomeres and aging. Physiol. Rev..

[19] Proctor C.J., Kirkwood T.B. (2002). Modelling telomere shortening and the role of oxidative stress. Mech. Ageing Dev..

[20] Von Zglinicki T., Martin-Ruiz C.M. (2005). Telomeres as biomarkers for ageing and age-related diseases. Curr. Mol. Med..

[21] Sidorov I., Kimura M., Yashin A., Aviv A. (2009). Leukocyte telomere dynamics and human hematopoietic stem cell kinetics during somatic growth. Exp. Hematol..

[22] Blackburn E.H. (2000). Telomere states and cell fates. Nature.

[23] Blasco M.A. (2007). Telomere length, stem cells and aging. Nat. Chem. Biol..

[24] Mirabello L., Huang W.-Y., Wong J.Y., Chatterjee N., Reding D., Crawford E.D., De Vivo I., Hayes R.B., Savage S.A. (2009). The association between leukocyte telomere length and cigarette smoking, dietary and physical variables, and risk of prostate cancer. Aging Cell.

[25] Morlà M., Busquets X., Pons J., Sauleda J., MacNee W., Agusti A. (2006). Telomere shortening in smokers with and without COPD. Eur. Respir. J..

[26] Von Zglinicki T. (2002). Oxidative stress shortens telomeres. Trends Biochem. Sci..

[27] Shammas M.A. (2011). Telomeres, lifestyle, cancer, and aging. Curr. Opin. Clin. Nutr. Metab. Care.

[28] Kim N.W., Piatyszek M.A., Prowse K.R., Harley C.B., West M.D., Ho P.L.C., Coviello G.M., Wright W.E., Weinrich S.L., Shay J.W. (1994). Specific association of human telomerase activity with immortal cells and cancer. Science.

[29] Barthel F.P., Wei W., Tang M., Martinez-Ledesma E., Hu X., Amin S.B., Akdemir K.C., Seth S., Song X., Wang Q. (2017). Systematic analysis of telomere length and somatic alterations in 31 cancer types. Nat. Genet..

[30] Benetos A., Gardner J.P., Zureik M., Labat C., Xiaobin L., Adamopoulos C., Temmar M., Bean K.E., Thomas F., Aviv A. (2004). Short Telomeres Are Associated With Increased Carotid Atherosclerosis in Hypertensive Subjects. Hypertension.

[31] Brouilette S., Singh R.K., Thompson J.R., Goodall A.H., Samani N.J. (2003). White cell telomere length and risk of premature myocardial infarction. Arterioscler. Thromb. Vasc. Biol..

[32] Panossian L.A., Porter V.R., Valenzuela H.F., Zhu X., Reback E., Masterman D., Cummings J.L., Effros R.B. (2003). Telomere shortening in T cells correlates with Alzheimer’s disease status. Neurobiol. Aging.

[33] Weng X., Zhang H., Kan M., Ye J., Liu F., Wang T., Deng J., Tan Y., He L., Liu Y. (2015). Leukocyte telomere length is associated with advanced age-related macular degeneration in the Han Chinese population. Exp. Gerontol..

[34] Sunness J.S., Rubin G.S., Applegate C.A., Bressler N.M., Marsh M.J., Hawkins B.S., Haselwood D. (1997). Visual Function Abnormalities and Prognosis in Eyes with Age-related Geographic Atrophy of the Macula and Good Visual Acuity. Ophthalmology.

[35] Lindblad A.S., Lloyd P.C., E Clemons T., Gensler G.R., Ferris F., Klein R., Armstrong J.R. (2009). Change in Area of Geographic Atrophy in the Age-Related Eye Disease Study: AREDS report number 26. Arch. Ophthalmol..

[36] Liutkeviciene R., Vilkeviciute A., Streleckiene G., Kriauciuniene L., Chaleckis R., Deltuva V.P. (2017). Associations of cholesteryl ester transfer protein (CETP) gene variants with predisposition to age-related macular degeneration. Gene.

[37] Gedvilaite G., Vilkeviciute A., Kriauciuniene L., Banevičius M., Liutkeviciene R. (2020). The relationship between leukocyte telomere length and TERT, TRF1 single nucleotide polymorphisms in healthy people of different age groups. Biogerontology.

[38] Blackburn E.H. (2010). Telomeres and telomerase: The means to the end (Nobel lecture). Angew. Chem. Int. Ed. Engl..

[39] Immonen I., Seitsonen S., Saionmaa O., Fyhrquist F. (2013). Leucocyte telomere length in age-related macular degeneration. Acta Ophthalmol..

[40] Lee J.H., Anver M., Kost-Alimova M., Protopopov A., DePinho R.A., Rane S.G. (2014). Telomere dysfunction suppresses multiple endo-crine neoplasia in mice. Genes Cancer.

[41] Malaspina D., Dracxler R., Walsh-Messinger J., Harlap S., Goetz R.R., Keefe D., Perrin M.C. (2014). Telomere length, family history, and paternal age in schizophrenia. Mol. Genet. Genom. Med..

[42] Müezzinler A., Zaineddin A.K., Brenner H. (2013). A systematic review of leukocyte telomere length and age in adults. Ageing Res. Rev..

[43] Arai Y., Martin-Ruiz C.M., Takayama M., Abe Y., Takebayashi T., Koyasu S., Suematsu M., Hirose N., von Zglinicki T. (2015). Inflammation, But Not Telomere Length, Predicts Successful Ageing at Extreme Old Age: A Longitudinal Study of Semi-supercentenarians. eBioMedicine.

[44] Goglin S.E., Farzaneh-Far R., Epel E.S., Lin J., Blackburn E.H., Whooley M.A. (2016). Change in leukocyte telomere length predicts mor-tality in patients with stable coronary heart; disease from the heart and soul study. PLoS ONE.

[45] Mons U., Müezzinler A., Schöttker B., Dieffenbach A.K., Butterbach K., Schick M., Peasey A., De Vivo I., Trichopoulou A., Boffetta P. (2017). Leukocyte Telomere Length and All-Cause Mortality, Cardiovascular Disease, and Cancer Mortality: Results from Individual-Participant-Data Meta-Analysis of 2 Large Prospective Cohort Studies. Am. J. Epidemiol..

[46] Kong C.M., Lee X.W., Wang X. (2013). Telomere shortening in human diseases. FEBS J..

[47] Armanios M.Y., Chen J.J.-L., Cogan J.D., Alder J.K., Ingersoll R.G., Markin C., Lawson W.E., Xie M., Vulto I., Phillips J.A. (2007). Telomerase Mutations in Families with Idiopathic Pulmonary Fibrosis. New Engl. J. Med..

[48] Nilsson P.M., Tufvesson H., Leosdottir M., Melander O. (2013). Telomeres and cardiovascular disease risk: An update 2013. Transl. Res..

[49] Adnot S., Amsellem V., Boyer L., Marcos E., Saker M., Houssaini A., Kebe K., Dagouassat M., Lipskaia L., Boczkowski J. (2015). Telomere Dysfunction and Cell Senescence in Chronic Lung Diseases: Therapeutic Potential. Pharmacol. Ther..

[50] Valdes A.M., Richards J.B., Gardner J.P., Swaminathan R., Kimura M., Xiaobin L., Aviv A., Spector T.D. (2007). Telomere length in leukocytes correlates with bone mineral density and is shorter in women with osteoporosis. Osteoporos. Int..

[51] Deleidi M., Jaggle M., Rubino G. (2015). Immune aging, dysmetabolism, and inflammation in neurological diseases. Front Neurosci..

[52] Boccardi V., Pelini L., Ercolani S., Ruggiero C., Mecocci P. (2015). From cellular senescence to Alzheimer’s disease: The role of telomere shortening. Ageing Res. Rev..

[53] Blasco M.A. (2005). Telomeres and human disease: Ageing, cancer and beyond. Nat Rev Genet..

[54] Zarbin M.A. (2004). Current Concepts in the Pathogenesis of Age-Related Macular Degeneration. Arch. Ophthalmol..

[55] Drigeard Desgarnier M.C., Zinflou C., Mallet J.D., Gendron S.P., Méthot S.J., Rochette P.J. (2016). Telomere Length Measurement in Different Ocular Structures: A Potential Implication in Corneal Endothelium Pathogenesis. Investig. Ophthalmol. Vis. Sci..

[56] Bell W.R., Meeker A.K., Rizzo A., Rajpara S., Rosenthal I.M., Bellver M.F., Domingo S.A., Zhong X., Barber J.R., Joshu C.E. (2019). A unique telomere DNA expansion phenotype in human retinal rod photoreceptors associated with aging and disease. Brain Pathol..

[57] Bhutto I., Lutty G. (2012). Understanding age-related macular degeneration (AMD): Relationships between the photoreceptor/retinal pigment epithelium/Bruch’s membrane/choriocapillaris complex. Mol. Asp. Med..

[58] Blasiak J. (2020). Senescence in the pathogenesis of age-related macular degeneration. Cell. Mol. Life Sci..

[59] Blasiak J., Szczepanska J., Fila M., Pawlowska E., Kaarniranta K. (2021). Potential of Telomerase in Age-Related Macular Degeneration—Involvement of Senescence, DNA Damage Response and Autophagy and a Key Role of PGC-1α. Int. J. Mol. Sci..

[60] Pallini R., Sorrentino A., Pierconti F., Maggiano N., Faggi R., Montano N., Maira G., Larocca L.M., Levi A., Falchetti M.L. (2006). Telomerase inhibition by stable RNA interference impairs tumor growth and angiogenesis in glioblastoma xenografts. Int. J. Cancer.

[61] Martin-Ruiz C.M., Gussekloo J., van Heemst D., von Zglinicki T., Westendorp R.G. (2005). Telomere length in white blood cells is not associated with morbidity or mortality in the oldest old: A population-based study. Aging Cell.

[62] Campa D., Matarazzi M., Greenhalf W., Bijlsma M., Saum K.-U., Pasquali C., Van Laarhoven H., Szentesi A., Federici F., Vodička P. (2019). Genetic determinants of telomere length and risk of pancreatic cancer: A PANDoRA study. Int. J. Cancer.

[63] Huang F.W., Hodis E., Xu M.J., Kryukov G.V., Chin L., Garraway L.A. (2013). Highly Recurrent TERT Promoter Mutations in Human Melanoma. Science.

[64] Horn S., Figl A., Rachakonda P.S., Fischer C., Sucker A., Gast A., Kadel S., Moll I., Nagore E., Hemminki K. (2013). TERT Promoter Mutations in Familial and Sporadic Melanoma. Science.

[65] Zhang R., Zhao J., Xu J., Liu F., Xu Y., Bu X., Dai C., Song C. (2015). Genetic variations in the TERT and CLPTM1L gene region and gastrointestinal stromal tumors risk. Oncotarget.

[66] Yuan X., Cheng G., Yu J., Zheng S., Sun C., Sun Q., Li K., Lin Z., Liu T., Li P. (2017). The TERT promoter mutation incidence is modified by germline TERT rs2736098 and rs2736100 polymorphisms in hepatocellular carcinoma. Oncotarget.

[67] Zhou M., Jiang B., Xiong M., Zhu X. (2018). Association Between TERT rs2736098 Polymorphisms and Cancer Risk-A Meta-Analysis. Front. Physiol..

[68] Yin J., Li Y., Yin M., Sun J., Liu L., Qin Q., Li X., Long L., Nie S., Wei S. (2012). TERT-CLPTM1L Polymorphism rs401681 Contributes to Cancers Risk: Evidence from a Meta-Analysis Based on 29 Publications. PLoS ONE.

[69] Hosgood H.D., Cawthon R., He X., Chanock S., Lan Q. (2009). Genetic variation in telomere maintenance genes, telomere length, and lung cancer susceptibility. Lung Cancer.

[70] Wu Q., Han D., Zhang J., Li X. (2019). Expression of telomere repeat binding factor 1 and TRF2 in Alzheimer’s disease and correlation with clinical parameters. Neurol. Res..

[71] Shi J.-M., Huang H., Chen Q.-F., Lin M.-F. (2006). A study of the relationship between expression level of TRF1 protein and telomerase activity in human acute leukemia. J. Zhejiang Univ. Sci. B.

[72] Lin X., Gu J., Lu C., Spitz M.R., Wu X. (2006). Expression of Telomere-Associated Genes as Prognostic Markers for Overall Survival in Patients with Non–Small Cell Lung Cancer. Clin. Cancer Res..

